# Phototrophic biofilm assembly in microbial-mat-derived unicyanobacterial consortia: model systems for the study of autotroph-heterotroph interactions

**DOI:** 10.3389/fmicb.2014.00109

**Published:** 2014-04-07

**Authors:** Jessica K. Cole, Janine R. Hutchison, Ryan S. Renslow, Young-Mo Kim, William B. Chrisler, Heather E. Engelmann, Alice C. Dohnalkova, Dehong Hu, Thomas O. Metz, Jim K. Fredrickson, Stephen R. Lindemann

**Affiliations:** ^1^Biological Sciences Division, Fundamental and Computational Sciences Directorate, Pacific Northwest National LaboratoryRichland, WA, USA; ^2^Chemical, Biological, and Physical Sciences Division, National Security Directorate, Pacific Northwest National LaboratoryRichland, WA, USA; ^3^Scientific Resources Division, William R. Wiley Environmental Molecular Sciences Laboratory, Pacific Northwest National LaboratoryRichland, WA, USA

**Keywords:** phototrophic biofilm model system, microbial diversity, primary succession, real-time PCR, community metabolomics, confocal microscopy, image analysis, microcosm

## Abstract

Microbial autotroph-heterotroph interactions influence biogeochemical cycles on a global scale, but the diversity and complexity of natural systems and their intractability to *in situ* manipulation make it challenging to elucidate the principles governing these interactions. The study of assembling phototrophic biofilm communities provides a robust means to identify such interactions and evaluate their contributions to the recruitment and maintenance of phylogenetic and functional diversity over time. To examine primary succession in phototrophic communities, we isolated two unicyanobacterial consortia from the microbial mat in Hot Lake, Washington, characterizing the membership and metabolic function of each consortium. We then analyzed the spatial structures and quantified the community compositions of their assembling biofilms. The consortia retained the same suite of heterotrophic species, identified as abundant members of the mat and assigned to *Alphaproteobacteria*, *Gammaproteobacteria*, and *Bacteroidetes.* Autotroph growth rates dominated early in assembly, yielding to increasing heterotroph growth rates late in succession. The two consortia exhibited similar assembly patterns, with increasing relative abundances of members from *Bacteroidetes* and *Alphaproteobacteria* concurrent with decreasing relative abundances of those from *Gammaproteobacteria*. Despite these similarities at higher taxonomic levels, the relative abundances of individual heterotrophic species were substantially different in the developing consortial biofilms. This suggests that, although similar niches are created by the cyanobacterial metabolisms, the resulting webs of autotroph-heterotroph and heterotroph-heterotroph interactions are specific to each primary producer. The relative simplicity and tractability of the Hot Lake unicyanobacterial consortia make them useful model systems for deciphering interspecies interactions and assembly principles relevant to natural microbial communities.

## Introduction

Comprehending interspecies interactions is critical to understanding niche formation and maintenance of diversity in microbial communities. However, elucidation of these interactions has been hampered by the complexity of natural microbial communities and the difficulty of assaying the behavior of the individual species that compose them (Wintermute and Silver, 2010). Cyanobacteria are frequently associated with heterotrophic bacteria in nature (Paerl, 1977), and geological evidence of interspecies interactions between cyanobacteria and heterotrophs dates to 440 m.y. ago (Tomescu et al., 2008). The cohesiveness of such interactions is illustrated by the paucity of axenic cyanobacterial strains, despite persistent attempts to isolate them from their consorts (Ferris and Hirsch, 1991). A unicyanobacterial consortium (UCC) is a phototrophic community in which one such cyanobacterium, serving as the sole or primary autotroph, supplies photosynthetically-derived carbon, and oxygen to one or more heterotrophs. The heterotrophs, in turn, can promote cyanobacterial growth by providing key metabolites and scavenging wastes (Paerl and Pinckney, 1996; Paerl et al., 2000). In this way, a consortium's expanded and compartmentalized metabolic capacity synergistically improves resource utilization efficiency over that of its individual members.

Mutualistic, cyclical exchanges of metabolites between cyanobacteria and their heterotrophic consorts may involve intimate physical interactions; for example, carbon and nitrogen exchange between *Anabaena* sp. SSM-00 and epibiont *Rhizobium* sp. WH2K promotes specific adherence of the epibionts to the cyanobacterial heterocysts (Stevenson and Waterbury, 2006; Behrens et al., 2008; Stevenson et al., 2011). Many cyanobacteria grow more efficiently in co-cultivation with heterotrophic bacteria than in axenic cultures, especially when heterotrophic remineralization of organic carbon alleviates cyanobacterial carbon limitation (Lupton and Marshall, 1981; Schiefer and Caldwell, 1982; Morris et al., 2008; Abed, 2010; Hayashi et al., 2011; Shen et al., 2011). Specific, highly-adapted interspecies interactions improve nutrient (Paerl, 1977; Paerl and Kellar, 1978; Lupton and Marshall, 1981; Li et al., 2010; Roe et al., 2012; Van Mooy et al., 2012) and vitamin (Gordon et al., 1969; Kazamia et al., 2012; Xie et al., 2013) acquisition by cyanobacteria and eukaryotic microalgae in consortia. Because such microbial photoautotroph-heterotroph consortial relationships are pervasive, interspecies interactions occurring within these consortia exert globally-significant impacts upon biogeochemical cycles (Paerl and Pinckney, 1996; Ferrier et al., 2002; Grossart et al., 2006; Hmelo et al., 2012).

As compact, laminated microbial consortia, microbial mats promote functional integration and interaction between diverse community members through their complex spatial organization. Though the community diversity (e.g., Bolhuis and Stal, 2011) and functional capacity (e.g., Klatt et al., 2011, 2013) of cyanobacterial mat communities have been extensively investigated, few of these studies have examined mats with any temporal resolution (Ferris and Ward, 1997; Yannarell et al., 2006, 2007; Lacap et al., 2007; Bolhuis and Stal, 2011; Hegler et al., 2012) and even fewer incorporate any evaluation of the primary succession of these mats (Pinckney et al., 1995; Armitage et al., 2012). Further, only Armitage and coworkers incorporated any characterization of the communities' functional capacities. Therefore, mechanisms by which phototrophic mat communities assemble, especially with respect to the recruitment and maintenance of phylogenetic diversity (Kassen and Rainey, 2004; Fierer et al., 2010) and functional capacity (Johnson et al., 2012), remain poorly understood.

Hot Lake is a meromictic, hypersaline lake that contains extreme concentrations of magnesium sulfate and seasonally harbors a benthic phototrophic microbial mat (Lindemann et al., 2013). The lake experiences significant dynamicity in major environmental parameters over 1 year, illustrated by a nearly tenfold variation in salinity over 2011. The mat community assembles and disassembles annually, colonizing bare sediments in spring, reaching maximal diversity over the summer, and displaying evidence of disassembly by mid-autumn. Despite the significant environmental variability, the mat maintains a relatively stable community structure. Throughout the seasonal cycle, ~80% of amplicon sequences from the V4 region of the 16S rRNA gene (*rrnA*) can be attributed to members of phyla *Cyanobacteria*, *Proteobacteria* (specifically, classes *Alphaproteobacteria* and *Gammaproteobacteria*), and *Bacteroidetes*. This stability is also generally observed at the greater taxonomic resolution of an operational taxonomic unit (OTU), defined at a minimum level of 97% average identity in 16S rRNA gene sequence. This suggests that a robust network of interspecies interactions between the mat's cyanobacteria and heterotrophs may impart community resistance to significant environmental variation. However, stochastic shifts in environmental conditions and high species richness (Lindemann et al., 2013) make it challenging to uncover the ecological forces governing cyanobacterium-heterotroph interactions *in situ*.

Here we describe two unicyanobacterial consortia cultivated from the Hot Lake mat as model systems in which to study the community structure and metabolic function of an assembling phototrophic biofilm. To the best of our knowledge, this study is the first since that of Gordon et al. (1969) to quantify changes in the abundance of individual heterotrophic species in assembling phototrophic biofilms within a closed microecosystem (*sensu* Gordon, et al.). As their study was performed before the advent of molecular techniques for microbial detection and enumeration, Gordon and coworkers were limited to quantifying populations of cultivable organisms and were therefore unable to estimate the diversity of potential colonizers in the microecosystem. Next-generation sequencing now permits greatly-expanded characterization of consortial membership, and species-resolved molecular quantitation allows the assessment of heterotroph abundance independent of cultivation. In this study, we define the membership and examine the phototroph-heterotroph succession of unicyanobacterial consortia derived from the Hot Lake microbial mat.

## Materials and methods

### Isolation and enrichment

Hot Lake is located near Oroville, WA at 48.973062°N, 119.476876°W. Cyanobacteria and their heterotrophic consorts were isolated from a benthic mat sample collected from the lake on July 7, 2011 at a depth of 35 cm in water of 29.6°C and 117.9 g/L of total dissolved solids (Lindemann et al., 2013). Enrichment cultures were generated by subdividing the mat lamina and streaking a subsample of the green layer onto BG-11 plates (Stanier et al., 1971) buffered with 10 mM TES pH 8.0 and amended with 400 mM MgSO_4_, 80 mM Na_2_SO_4_, 20 mM KCl, 1 mM NaHCO_3,_ hereafter referred to as Hot Lake autotroph (HLA) medium. HLA was amended with 1× Wolfe's vitamins (Wolin et al., 1963) for initial isolation. Solid medium was generated through the addition of 10 g/L triple-washed agar (Thermo Fisher Scientific, Waltham, MA). Unless noted, all chemicals and reagents were purchased from Sigma-Aldrich, Saint Louis, MO. Plates were incubated at room temperature (~23°C) under a fluorescent grow light (General Electric PL/AQ, Fairfield, CT) with a continuous photon flux of 20 μE/m^2^/s. No exogenous sources of organic carbon were added. Two morphologically-distinct cyanobacteria that grew on the plate were resuspended in HLA broth and physically isolated with a micropipette under a dissecting microscope. Each filament, including its co-isolated heterotrophs, was inoculated into sterile HLA broth and cultivated, yielding two unicyanobacterial consortia, UCC-A and UCC-O. The consortia were thereafter maintained continuously, with passaging performed every 4 weeks at a dilution of 1:100 into 100 mL of HLA broth in 250 mL borosilicate-glass Erlenmeyer flasks under the cultivation conditions described above. The enrichment cultures were physically homogenized prior to subculturing by dividing the cultures into sterile 50 mL conical vials with sterile 3 mm glass beads and vigorously shaking the tubes by hand for ~15 s to disrupt the biofilms. Heterotrophs were isolated from the Hot Lake mat or enrichment cultures by streaking a portion of a homogenized sample onto HLA plates supplemented with 0.25% yeast extract and 5 mM NH_4_Cl, henceforth referred to as Hot Lake Heterotroph (HLH) medium, and incubating at room temperature in the dark. Single colonies were picked and streaked at least five times to obtain pure heterotroph cultures. Purity was verified by light microscopy and *rrnA* sequencing.

### Cultivation for biofilm assembly experiments

**Figure 1:**
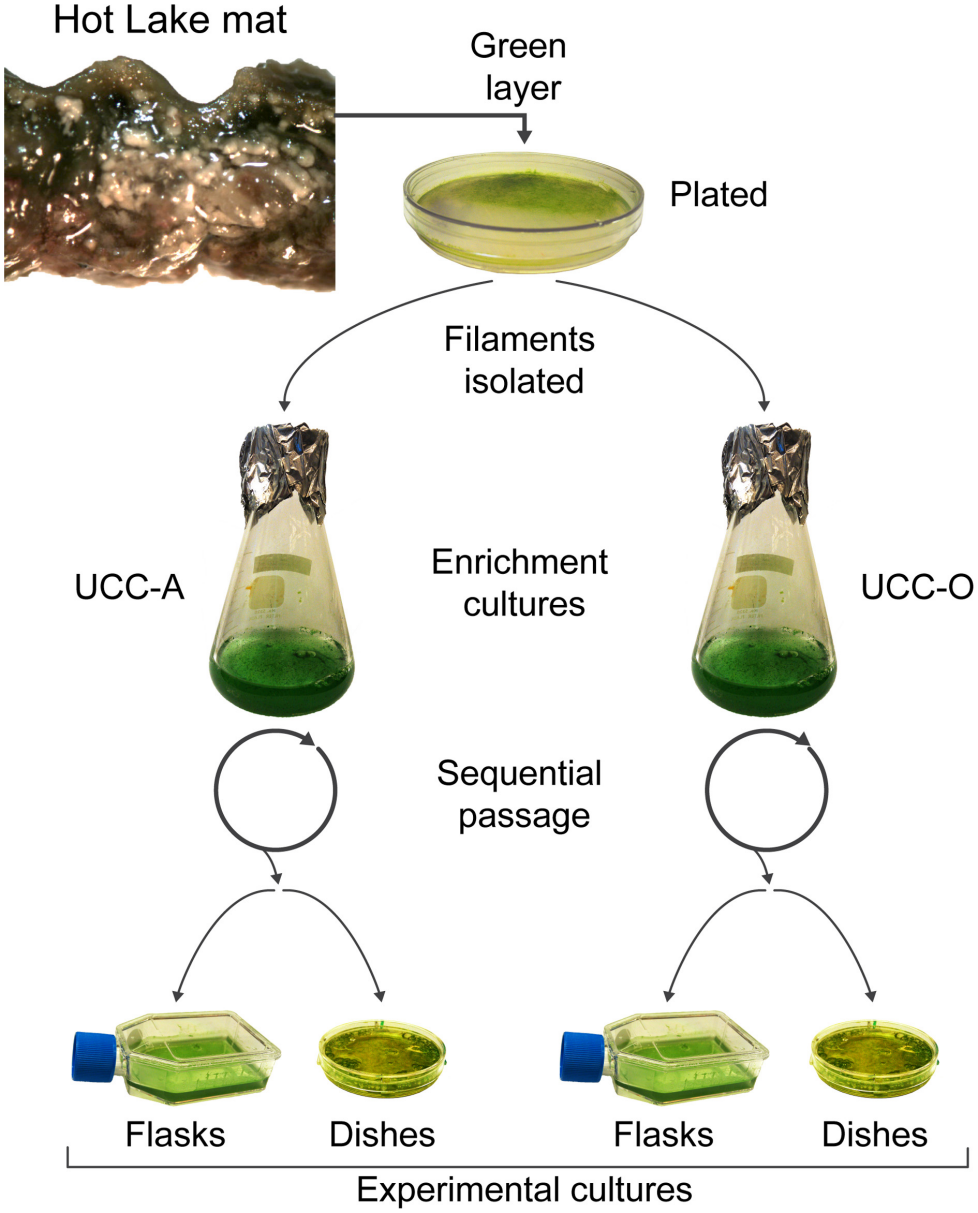
**Isolation and cultivation of the unicyanobacterial consortia**. Biomass from a sample of the green layer of the Hot Lake mat was streaked onto plates. Two morphologically-distinct cyanobacterial filaments were isolated and inoculated into broth enrichment cultures. These enrichment cultures were sequentially passaged and served as inocula for experimental cultures. Experimental cultures were grown in tissue culture flasks for metabolomic, composition (dry weight, total protein, chlorophyll *a*, cell counts), and real-time PCR analyses. Glass-bottom dishes were used for microscopic analysis of the biofilms.

UCC-A and UCC-O enrichment cultures were harvested and homogenized as described above, and cultures were propagated for biofilm assembly experiments in triplicate T25 tissue culture flasks with vented caps (Costar, Corning, Corning, NY) by adding 1:50 inoculum of the enrichment cultures into 10 mL of HLA broth (hereafter referred to as experimental cultures). Experimental cultures developed benthic biofilms as they were incubated for 28 days under a continuous photon flux of 35 μE/m^2^/s (General Electric PL/AQ) at room temperature (~23°C). For confocal microscopy of biofilms during assembly studies, consortia were cultivated in FluoroDish 60 mm glass-bottom tissue culture dishes (World Precision Instruments, Sarasota, FL). Dishes for microscopic analysis were rehydrated weekly with sterile water to restore the culture volume lost to evaporation. The process by which enrichment and experimental cultures were generated is highlighted in Figure 1.

### Electron microscopy

For scanning electron microscopy (SEM) analysis, cell suspensions were adhered to 0.2 μm pore polycarbonate track-etched membranes and fixed in 2.5% glutaraldehyde (Electron Microscopy Sciences (EMS), Hatfield, PA) for 2 h. Cells were washed three times in 0.1 M sodium cacodylate buffer (EMS) and gradually dehydrated in an ethanol series (25, 33, 50, 75, 90%), followed by three washes in 100% ethanol (15 min each). After dehydration, the cells were processed in the automatic critical point dryer (CPD) Autosamdri-815 (Tousimis, Rockville, MD), with CO_2_ as a transitional fluid. The CPD-processed membranes were mounted on aluminum SEM stubs and sputter-coated with carbon. Cells were imaged with a Helios 600 Nanolab dual-beam microscope (FEI, Hillsboro, OR) at 2 kV.

For transmission electron microscopy (TEM) analysis, cell suspensions were pelleted by centrifugation (15,000 × *g*, 4°C, 10 min), fixed, and dehydrated as described for SEM. After the last ethanol wash, samples were gradually infiltrated in Spurr's low-viscosity embedding media (EMS). After polymerization at 60°C for 24 h, the hardened resin blocks were sectioned on a Leica EM UC6 Ultramicrotome using a 45° diamond knife (Diatome, Hatfield, PA). Seventy-nanometer sections were post-stained with 2% uranyl acetate and Reinold's lead citrate (seven and 3 min, respectively) and imaged in a Tecnai T-12 TEM (FEI) at 120 kV.

### Nucleic acids extraction

Total DNA was extracted from 1 mL of homogenized culture using the MasterPure Complete DNA and RNA Purification Kit (Epicentre, Madison, WI). Extractions were conducted according to the manufacturer's protocols for cell samples and precipitation of total DNA. The DNA concentration was measured with the Qubit HS assay (Life Technologies, Carlsbad, CA).

### Clone library construction, sequencing, and processing

UCC-A and UCC-O near-full-length PCR products targeting *rrnA* were generated and cloned as previously described (Lindemann et al., 2013). Briefly, genomic DNA (gDNA) harvested from UCC-A and UCC-O was amplified with PCR primers 27F (5′-AGAGTTTGATCMTGGCTCAG-3′) and 1492R (5′-GGYTACCTTGTTACGACTT-3′) (Lane et al., 1991). PCR was performed using the Phusion High-Fidelity PCR Kit (New England BioLabs, Ipswitch, MA) in HF Buffer and 3% dimethyl sulfoxide according to the manufacturer's instructions. The annealing temperature was 55°C for 27 cycles. Products were cloned using the Zero Blunt TOPO PCR Cloning Kit (Life Technologies), and inserts were sequenced by Sanger dideoxy chain-termination sequencing from the SP6 and T7 promoter regions by Functional Biosciences (Madison, WI). The ContigExpress algorithm of Vector NTi Advance v. 11.0 (Life Technologies) was used to trim sequence ends, check for vector contamination, and assemble contigs. Contigs were aligned in mothur v. 1.29 (Schloss et al., 2009) using the mothur-formatted SILVA-based bacterial reference alignment (http://www.mothur.org/w/images/9/98/Silva.bacteria.zip, updated April 22, 2012). The aligned sequences were clustered to account for the expected error of a Phred score of 20 (1%, allowing twelve differences across the alignment). Chimeras were checked using the UCHIME algorithm in mothur 1.29 (Edgar et al., 2011) and removed. The remaining sequences were used as a reference to map Itag sequences (see below). Near-full-length clones that mapped >0.1% of the Itag sequences were manually examined for chimeras and submitted to GenBank (see Table S1, for accession numbers).

### Itag sequencing, processing and analysis

To evaluate the complexity of the consortia, gDNA from three-week-old UCC-A and UCC-O enrichment cultures was sequenced for short 16S rRNA tag (Itags) analysis. Sequencing was done on an Illumina MiSeq instrument at the Joint Genome Institute, Walnut Creek, CA as described previously (Lindemann et al., 2013). Briefly, for each sample, three separate 16S rRNA amplification reactions were performed using the V4 forward primer (515F) and V4 reverse primer (806R) with 0–3 random bases and the Illumina sequencing primer binding site (Caporaso et al., 2010). A total of 149,869 (70,082 for UCC-A and 79,787 for UCC-O) barcoded paired-end reads were obtained after computational removal of PhiX and contaminant reads (reads containing Illumina adapters); these were paired-end assembled using FLASH software (Magoč and Salzberg, 2011).

Sequences were processed as previously described using mothur v. 1.31 (Schloss et al., 2009; Kozich et al., 2013) according to the online MiSeq protocol (http://www.mothur.org/wiki/MiSeq_SOP [accessed 9/12/13]). Briefly, those with ambiguities or those shorter than 251 nts were removed. The remaining sequences were aligned to the SILVA-based bacterial reference alignment to which near-full-length sequences from the Hot Lake mat had been added (Lindemann et al., 2013). Afterwards, they were screened to remove those that did not align to positions 13,871–23,444 of the reference alignment, preclustered, and checked for chimeras using UCHIME (mothur 1.31 in self-referential mode). Sequences were then classified using a Wang approach and the default settings of classify.seq with the Ribosome Database Project training set v. 9 (updated March 20, 2012 and formatted for mothur). Those of unknown classification at the kingdom level were removed. UCC-O sequences were subsampled to the size of the smaller group (UCC-A; *n* = 29,085 sequences) and clustered into OTUs using an average neighbor algorithm with a 3% cutoff, which were classified using the sequence classifications above. OTUs with a relative abundance of less than 0.025% were excluded from the analysis, as many OTUs with relative abundances below this value were found to have chimeric properties when examined manually. However, we cannot exclude the possibility that real but rare members of our consortia were represented in the long tail of low-abundance sequences produced by amplicon sequencing.

### Short-read mapping and phylogeny reconstruction

Unique Itag reads were mapped to near-full-length *rrnA* sequences from the consortia clone libraries using the nucmer algorithm in MUMmer v. 3.23 (Kurtz et al., 2004). An Itag match is defined as sharing at least 99% sequence identity with a clone sequence over a minimum length of 243 nts. Phylogeny reconstruction was performed using aligned near-full-length clone sequences with neighbor-joining and maximum likelihood algorithms over 1,410 positions of the alignment as previously described (Lindemann et al., 2013) in MEGA5.2 (Tamura et al., 2011). Phylogenies were tested using 1,000 bootstrap replications.

### Metabolomics analysis

Consortial biomass was harvested after 7, 14, 21, and 28 days of cultivation and dried *in vacuo*. All chemicals and reagents were purchased from Sigma-Aldrich unless otherwise noted. Dry biomass weight was recorded prior to resuspension with 200 μL of nanopure water to which was added a half volume of 0.1 mm zirconium/silica beads (Biospec, Bartlesville, OK). The biomass was homogenized by vortexing for 2 min, and metabolites were extracted with 800 μL of chloroform/methanol mixture (2:1, v/v). After centrifugation (15,000 × *g*, 4°C, 5 min) the aqueous layer was transferred to glass vials and dried *in vacuo*. Polar metabolites from microbial mat collected at Hot Lake on June 27, 2012 at 16:00 and frozen at −80°C were extracted using the same procedure. A mixture of fatty acid methyl esters (FAMEs; C_8_–C_28_) dissolved in hexane was used as a retention index standard.

Polar metabolite analysis was performed with an Agilent 7890A gas chromatograph coupled with a single quadrupole MSD 5975C mass spectrometer (Agilent Technologies, Santa Clara, CA). For untargeted analysis of polar metabolites, dried aqueous layers were chemically derivatized as previously described (Kim et al., 2011, 2013) and analyzed in duplicate. Samples were analyzed according to the method used to create the Agilent Fiehn Metabolomics Retention Time Locked (RTL) Library (Kind et al., 2009). An HP-5MS column (30 m × 0.25 mm × 0.25 μm; Agilent Technologies, Inc.) was used. The sample injection mode was splitless, and 1 μL of each sample was injected. The injection port temperature was held at 250°C throughout the analysis. The GC oven was held at 60°C for 1 min after injection, and the temperature was then increased to 325°C by 10°C/min, followed by a 5 min hold at 325°C (Kim et al., 2013). The helium gas flow rate was determined by the Agilent Retention Time Locking function based on analysis of deuterated myristic acid (Agilent Technologies) and was in the range of 0.45–0.5 mL/min. Data were collected over the mass range 50–550 m/z. The mixture of FAMEs (C8–C28) was analyzed once per day alongside the samples to standardize retention index alignment in subsequent data analysis.

Gas chromatography-mass spectrometry (GC-MS) raw data files were processed using MetaboliteDetector (Hiller et al., 2009). Briefly, retention indices (RI) of detected metabolites were calculated based on the analysis of the FAMEs mixture, followed by their chromatographic alignment across all analyses after deconvolution. Metabolites were then identified by matching GC-MS features (characterized by measured RI and mass spectra) to an augmented version of the Agilent Fiehn Metabolomics RTL Library (Kind et al., 2009). This library contains spectra and validated RI for over 700 metabolites and thus provides two metrics for confident metabolite identification. The NIST 08 GC-MS library was also used to cross-validate the spectral matching scores obtained using the Agilent library. All metabolite identifications were manually validated to eliminate false identifications. Raw GC-MS data is available via the Metabolights metabolomics data repository as study MTBLS75 (http://www.ebi.ac.uk/metabolights/index) (Haug et al., 2013).

### Total protein analysis

Total community protein was extracted with 300 μL of B-PER Reagent (Pierce, Thermo Fisher Scientific). Insoluble protein was pelleted by centrifugation (15,000 × *g*, 4°C, 10 min). Total protein and peptides were quantified with a BCA kit (Thermo Fisher Scientific) according to manufacturer directions using bovine serum albumin to prepare the standard curve.

### Chlorophyll quantification

Total chlorophyll was extracted and measured using a modification of the protocol reported by Tandeau de Marsac (Marsac, 1977). One mL of culture was resuspended in pure methanol and incubated in the dark at 4°C for 1 h. Insoluble biomass was pelleted by (15,000 × *g*, 4°C, 5 min) and absorption of the supernatant was measured at 655 nm on a Tecan Safire^1^ microplate reader (San Jose, CA). Chlorophyll *a* concentration (μg/mL) was calculated using a standard curve made from spinach-derived chlorophyll *a* (Sigma-Aldrich) in methanol.

### Flow cytometry

Cell counts were carried out in a BD Influx cell sorter (BD Biosciences, San Jose, CA) triggered on forward scatter. Homogenized samples of UCC biomass were incubated in disodium EDTA (50 mM final concentration) and further disrupted with two rounds of vortexing and one round of sonication (bath sonicator for 1 min) prior to passage 25 times through a 25 gauge needle. The sample was split in half, with a portion left untreated (unstained control) and the other stained with SYBR Gold (Life Technologies) according to manufacturer's instructions. The unstained control was used to set a gate for background fluorescence, and data acquired for the stained sample were used to set a gate for the SYBR Gold-stained population. One hundred μL of stained sample was passed through the instrument and counted. This number was multiplied by the percentage of SYBR-positive cells to get the total number of nucleic-acid-positive cells.

### Confocal microscopy and image analysis

For confocal laser scanning microscopy, cultures at 7, 14, 21, and 28 days of age were stained with SYBR Gold for 10 min and then placed onto a Leica DMI6000 microscope equipped with a CSU 10 Confocal scanning unit (Yokogawa Corporation of America, Sugar Land, TX). Thirty random fields in each culture were selected for imaging of both SYBR Gold (488 nm Ex; 413–485 nm Em) and chlorophyll *a* autofluorescence (642 nm Ex; 663–738 nm Em) with a Leica Plan APO 20/0.7 objective using a Coolsnap HQ^2^ (Photometrics, Tucson, AZ) controlled by MetaMorph (Molecular Devices, Sunnyvale, CA) software. Confocal Z-stacks of the cultures for each color were collected at a 0.5 μm step size over a range of 30 μm. The images were further processed with Volocity (Perkin Elmer, Waltham, MA).

The morphology of developing consortial biofilms was investigated by analyzing confocal laser scanning micrographs using Image Structure Analyzer-2 (ISA-2) software (Biofilm Research Group; Beyenal et al., 2004a,b) and modified ISA-2 functions, executed using MATLAB (64 bit Matlab v8.0.0.783, The MathWorks, Inc., Natick, MA). Specifically, ISA-2 functions were modified to quantitate total biomass, autotroph (cyanobacterial chlorophyll *a* autofluoresence), and heterotroph (SYBR Gold) images independently. Bitwise logic operation threshold was gated on the chlorophyll *a* and SYBR Gold fluorescence intensities. Calculated 2D and 3D parameters included biomass fraction, biovolume, biofilm thickness, and cyanobacterium width. Biovolume is defined as the volume of cellular biomass within the consortial biofilm, and the biomass fraction is defined as the ratio of the surface area of cellular biomass to the field of view area. Each parameter, standard deviation, and confidence interval was calculated from 16 randomly-chosen locations on each of two biological replicates (a total of 32 image series for each time point) per UCC. Parameter outliers were detected using the outlier labeling rule (Hoaglin et al., 1986; Hoaglin and Iglewicz, 1987) based on multiplying the interquartile range by a conservative factor of 2.2.

### Real-time PCR

Dynamics of the community structure of both consortial biofilms were assessed using real-time PCR, targeting the *rrnA* genes of specific heterotrophic members (Table 1). All primers and probes were synthesized by Integrated DNA Technologies, Inc. (IDT, Coralville, IA). DNA concentrations were standardized to 1 ng from the four time points: 7, 14, 21, and 28 days. The PCR reaction was carried out in a total volume of 20 μL containing 5 μL of gDNA (1 ng/5 μL), 1 μL of PrimeTime qPCR Assay (20×, IDT), 10 μL of Applied Biosystems TaqMan Fast Universal No Amp master mix (2×, Life Technologies), and 4 μL of nuclease-free water. The reactions were amplified using the 45-min “fast” setting with the following cycling conditions: 95°C for 20 s, followed by 40 cycles of 95°C for 2 s, then 65°C for 30 s. Measurements were made in sextuplicate using submaster-mix preparation. The reactions and data analyses were performed using the ABI 7500 fast real-time PCR instrument and software. Specificity of the primer and probe sets was verified with purified gDNA from isolates in pure culture: *Idiomarinaceae* isolate HL-53, *Halomonas* sp. HL-48, *Marinobacter* sp. HL-58, *Marinobacter excellens* str. HL-55, and *Algoriphagus marincola* str. HL-49. The primer probe set for *Marinobacter* sp. HL-58 was cross-reactive with *M. excellens* str. HL-55 (data not shown), so both *Marinobacter* species were detected with this assay. Standard curves were performed for each target with template concentrations of 8 pg, 40 pg, 200 pg, 1 ng, and 5 ng/5 μL of genomic DNA in both consortia; 1 ng/5 μL fell within the linear range for each target reported as detected within each consortium. Standard curves are provided along with all *C*_*T*_-values in Table S3. Data are plotted as inverse *C*_*T*_ (1/*C*_*T*_) on a log_2_ scale.

**Table 1:** **Real-time PCR primers and probes**.

**Strain**	**Clone target**	**Primer/Probe**	**Sequence (5′–3′)^a^**
*Rhodobacteraceae*	ACL_P1H4	Forward	ATACTGACGCTGAGGTGCGAAAGT
		Probe	CCACACCGT/ZEN/AAACGATGAATGCCAGT
		Reverse	GTTAGGTGTGTCACCAAAGGGCAA
*Rhodobaca*	OCL_P2D11	Forward	ATACTGACGCTGAGGTGCGAAAGT
		Probe	AAACGATGA/ZEN/ATGCCAGACGTCGGCAA
		Reverse	TTAATCCGTTAGGTGTGACACCGA
*Oceanicaulis* sp. HL-87	OCL_P1H8	Forward	AGATATTCGGAGGAACACCAGAGG
		Probe	AAACGATGG/ZEN/ATGCTAGTTGTCGGGCA
		Reverse	TGCTTAATGCGTTAGCTGCGTCAC
*Rhizobiales*	ACL_P1B5	Forward	TATTCGCAAGAACACCAGTGGCGA
		Probe	ACGCCGTAA/ZEN/ACGATGAATGCCAGCTGTT
		Reverse	AATGCTCAAAGCGTTAACTGCGCC
*Idiomarinaceae* isolate HL-53	OCL_P1H2	Forward	TACCCTGGTAGTCCACGCTGTAAA
		Probe	CTAGTTGTC/ZEN/CGTTTCATAAACGAAGTGG
		Reverse	TCGACTTAGTGCGTTAGCTGCGTT
*Halomonas* sp. HL-48	ACL_P1H5	Forward	GAAAGCGTGGGTAGCAAACAGGAT
		Probe	TAGTCCACG/ZEN/CCGTAAACGATGTCGAC
		Reverse	TCGCGTTAACTTCGCCACAAAGTG
*Marinobacter* sp. HL-58	OCL_P1H5	Forward	ATAGGAAGGAACACCAGTGGCGAA
		Probe	ATACTGACA/ZEN/CTGAGGTGCGAAAGCGT
		Reverse	AAGACTTCAAGAGTCCCAACGGCT
*Algoriphagus marincola* str. HL-49	OCL_P2G9	Forward	TTACTCGCTGTTATGCCTTCGGGT
		Probe	AGCGGCCAA/ZEN/GCGAAAGCGTTAAGTAA
		Reverse	TTCCTTTGAGTTTCACCGTTGCCG
*Bacteroidetes*	OCL_P2A10	Forward	TGTATACTAGGTGTTGGCCCTGCT
		Probe	GGTCAGTGC/ZEN/TGCAGCTAACGCATTAA
		Reverse	GAGTTTCATCGTTGCCGACGTACT

a All probes were modified with 56-FAM (5′) and 3IABkFQ (3′) in addition to the internal ZEN modification.

## Results

### Characterization of unicyanobacterial consortia

**Figure 2:**
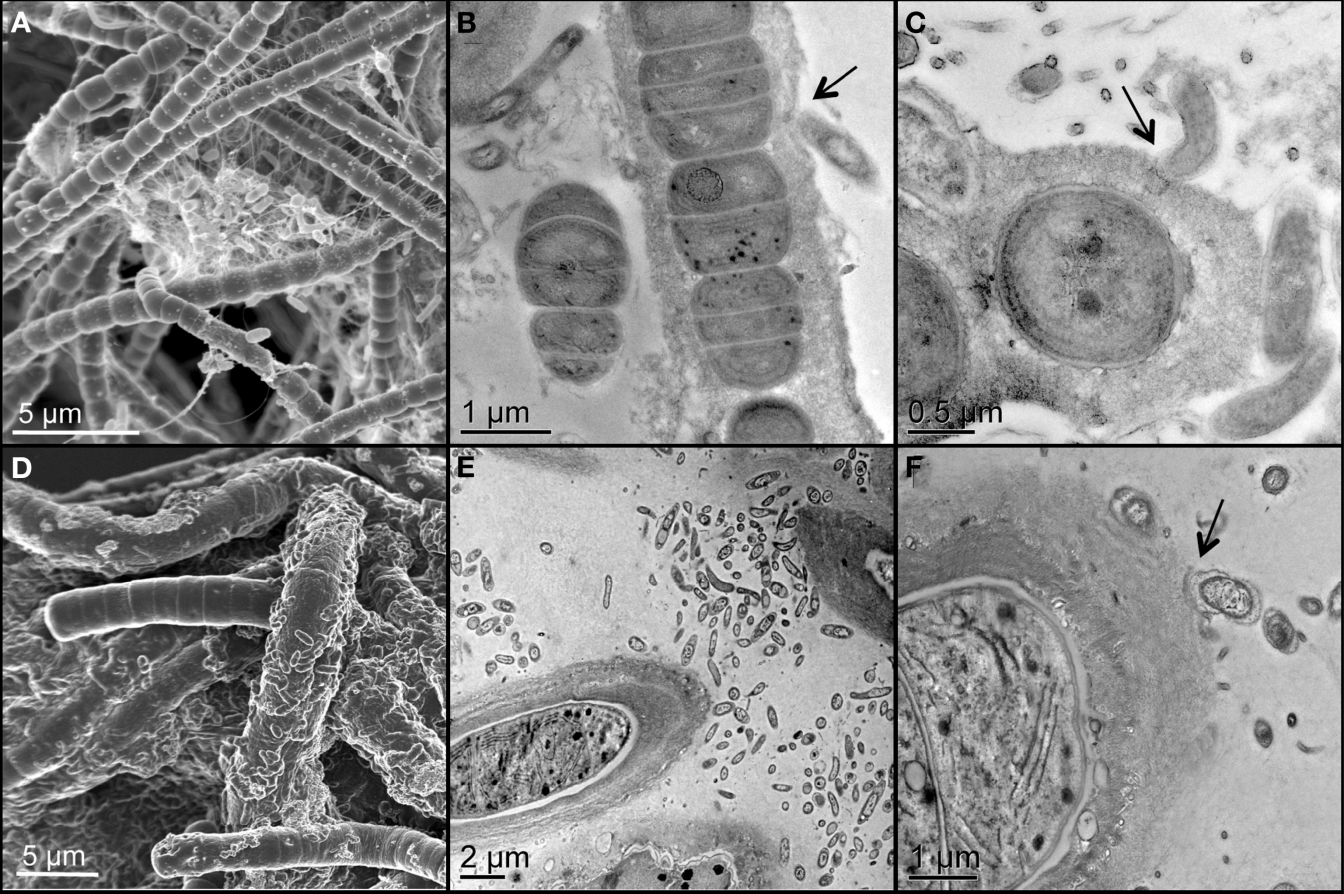
**Scanning and transmission electron micrographs of the consortia, UCC-A (A–C) and UCC-O (D–F)**. Filamentous cyanobacteria can be seen encased in low-electron-density exopolymeric substance (EPS) in close proximity with smaller, rod-shaped heterotrophic bacteria. Arrows highlight physical associations between cyanobacterial EPS and heterotrophs (**B,C,E,F**).

Two unicyanobacterial consortia (UCC-A and UCC-O) containing distinct species of cyanobacteria were isolated from a subsample of the Hot Lake phototrophic microbial mat. These consortia were passaged until they reached functional stability, as determined by proteomic and metabolomic analyses that showed no significant changes for greater than 6 months (peptide abundance biplot *R*^2^ > 0.99, data not shown). The cyanobacterium of UCC-O exhibited gliding motility, whereas that of UCC-A did not. Both cyanobacteria were filamentous with single, straight, non-branching trichomes (Figures 2A,D, Figure S1) encased in colorless sheaths composed of low-electron-density extracellular polymeric substance (EPS) (Figures 2B,C,E,F). The cyanobacterium in UCC-A had an average cell diameter of 1.72 μm (σ: 0.17 μm, confidence interval of the mean: 1.68–1.77 μm), and that in UCC-O had an average cell diameter of 3.33 μm (σ: 0.34 μm, confidence interval of the mean: 3.24–3.42 μm). Neither cyanobacterium was observed to form differentiated cells (heterocysts, necridia, calyptra, or aerotopes), though short filaments that could be undifferentiated hormogonia were observed in both cultures.

Both consortia maintained large heterotroph populations in broth lacking an exogenous organic carbon source. Heterotrophic epibionts were commonly associated physically with the EPS surrounding the filaments, though some filaments displayed few or no epibionts (Figure 2). The epibionts of UCC-A were frequently observed in clusters between cyanobacterial filaments, whereas those of UCC-O appeared more evenly-distributed along filaments (cf. Figures 2A,D).

### Community membership of the unicyanobacterial consortia

To assay consortial complexity, we performed amplicon sequencing across the V4 region (515F-806R) of the 16S rRNA gene (Itags) using gDNA harvested from enrichment cultures three weeks post-passage (Table 2). The Itag sequences were mapped to the corresponding region of near-full-length *rrnA* clone sequences derived from either the Hot Lake mat, the consortia, or a collection of strains isolated from the mat or consortia to gain increased phylogenetic resolution. Sequences identical to the representative sequence of all consortial OTUs were detected within the Hot Lake mat during the seasonal cycle of 2011 (Lindemann et al., 2013, Table S2).

**Table 2:** **Members of the consortia as determined by amplicon sequencing**.

**OTU**	**UCC-A^a^**	**UCC-O^a^**	**Representative sequence**	**% Reads matching^b^**	**Taxonomic assignment**	**Corresponding mat OTU^d^**	**Axenic representative^e^**
1	20,576	8,362	OCL_P1H2	94.6	*Idiomarinaceae*	535	HL-53
2	3,872	9,458	OCL_P2A10	86.5	*Bacteroidetes*	362	
3	146	6,168	ACL_P1B5	83.3	*Rhizobiales*	226	
4	193	4,741	OCL_P1H5	83.3	*Marinobacter*	255	HL-58
5	951	1,923	ACL_P1H5	89.0	*Halomonas*	241	HL-48
6	1,931	293	OCL_P2D11	72.1	*Rhodobaca*	237	
7	1	1,613	OCL_P2H12	81.0	Group XIII cyanobacterium	221	
8	444	484	OCL_P1H8	89.0	*Oceanicaulis*	325	HL-87
9	488	0	ACL_P2D9	97.1	Group IV cyanobacterium	344	
10	23	288	OCL_P1F4	75.9	*Marinobacter*	287	HL-55
11	48	75	No clone	-	*Erythrobacteraceae*	239	
12	2	12	No clone	-	*Rhodobacteraceae*	254	
13	31	53	HL7711_P4H5	86.9	*Roseibacterium*	225	
14	54	14	ACL_P1H4	96.6	*Rhodobacteraceae*	224	
15	39	6	HL7711_P3B12	77.8	*Rhodobacteraceae*	227	
16	17	12	OCL_P2G9	96.6	*Algoriphagus*	333	HL-49
17	0	24	HL-46^c^	83.3	*Porphyrobacter*	283	HL-46

**Figure 3:**
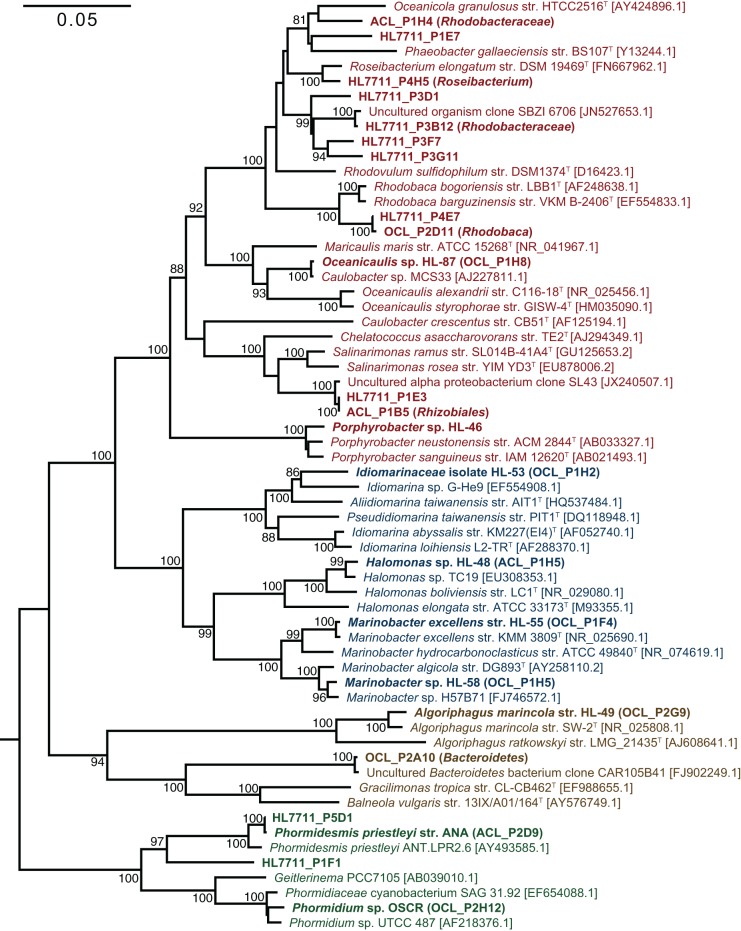
**Phylogenetic reconstruction of consortia members**. The phylogenetic tree was constructed by neighbor joining with 62 sequences over 1,410 aligned positions of the 16S rRNA gene. Sequences representing consortia members were selected from clone libraries of UCC-A (ACL), UCC-O (OCL), and the whole mat (HL7711) (Lindemann et al., 2013) and are bolded. Isolates in pure culture are designated by strain number (HL-XX). GenBank numbers for reference sequences are included in brackets and type strains for genera or species are denoted by a superscript T. Bootstrap values (1000 replications) exceeding 80% are indicated next to the node. A maximum-likelihood reconstruction of the phylogeny displayed the same topology as the one depicted. Clades are colored as follows: *Alphaproteobacteria*, red; *Gammaproteobacteria*; blue, *Cyanobacteria*, green; and *Bacteroidetes*, brown.

Itag reads of the V4 region of the rrnA gene were paired with matching near-full-length sequences to estimate the complexity of the consortia's memberships. Fourteen of the 15 heterotrophic OTUs were shared between the consortia, with UCC-A lacking any reads from Porphyrobacter sp. HL-46. Heterotrophic isolates representing seven of these OTUs were isolated into axenic culture. Sequences identical to those of the consortial OTUs have been previously identified within the Hot Lake mat community.

a Number of quality-filtered Itag reads composing each OTU segregated by consortium.

b The percent of Itag sequences in the OTU that share at least 99% sequence identity to the respective representative clone sequence.

c No clone was recovered, but a matching axenic culture was isolated from Hot Lake mat.

d Corresponding OTUs detected within the Hot Lake phototrophic mat over 2011, as described in Lindemann et al. (2013).

e Strain name of the cultured organism identical over the length of the Itag read to the most abundant read within an OTU (see Figure 3).

The primary producers of the consortia were a group XIII cyanobacterium (OTU 7) and a group IV cyanobacterium (OTU 9) in UCC-O and UCC-A, respectively. OTU 7 matched the near-full-length *rrnA* sequence of clone OCL_P2H12, which possessed 98.4% identity to *Phormidium* sp. UTCC 487 (Figure 3). Consequently, we designated this cyanobacterium *Phormidium* sp. OSCR. Twelve cyanobacterial OTUs of greater-than-0.01% average relative abundance were previously detected within the Hot Lake mat community; the sequence of *Phormidium* sp. OSCR is identical across the co-sequenced region to the second-most-abundant cyanobacterial OTU within the mat (Lindemann et al., 2013, data not shown). OTU 9, in UCC-A, matched clone ACL_P2D9 (Table 2), which shared 98.6% identity with *Phormidesmis priestleyi* ANT.LPR2.6 (basonym *Phormidium priestleyi*, Figure 3). Therefore, we designated this strain *Phormidesmis priestleyi* str. ANA. A solitary read from UCC-A clustered with those from *Phormidium* sp. OSCR, though with two mismatches to the representative sequence; this read was likely erroneously associated with UCC-A rather than UCC-O as no evidence of *Phormidium* sp. OSCR was detected within UCC-A by any other analyses. The observed morphological characterization of these cyanobacteria was consistent with that of their nearest neighbors (Figure S1, Casamatta et al., 2005; Komárek et al., 2009).

Despite distinct cyanobacteria serving as each consortium's primary producer, the heterotrophic memberships of UCC-A and UCC-O were nearly identical, sharing 14 of the 15 heterotrophic OTUs. The heterotrophs were classified within *Alphaproteobacteria, Gammaproteobacteria*, and *Bacteroidetes*, and represented genera known to contain marine and halophilic aerobes (Fuerst et al., 1993; Milford et al., 2000; Bowman et al., 2003; Ivanova et al., 2004; Jean et al., 2006; Suzuki et al., 2006; Boldareva et al., 2008). Seven of these heterotrophic strains were cultivated axenically (see Table 2 for connections between OTUs, cultures, and clone sequences used for phylogeny reconstruction in Figure 3). Class *Gammaproteobacteria* was represented by four organisms, all of which were isolated: *Marinobacter* sp. HL-58, *Marinobacter excellens* str. HL-55, *Halomonas* sp. HL-48 and an isolate that could not be classified below the family level, *Idiomarinaceae* isolate HL-53. *Bacteroidetes* was represented by the isolate *Algoriphagus marincola* str. HL-49, and a novel, deeply-branching organism that shared only ~85.0% identity to its nearest cultured relatives and could not be classified below the phylum level. Members of class *Alphaproteobacteria* comprised 9 of the 17 OTUs in the consortia, of which *Oceanicaulis* sp. HL-87 and *Porphyrobacter* sp. HL-46 were isolated. The uncultivated alphaproteobacteria included another member of *Erythrobacteraceae* that could not be assigned to a genus, a novel member of order *Rhizobiales*, and five organisms classified within *Rhodobacteraceae*. Two of these organisms could be assigned to genera within *Rhodobacteraceae* (*Rhodobaca* and *Roseibacterium*) that are known to contain aerobic anoxygenic phototrophs (AAPs) and may therefore be capable of photoheterotrophic growth (Milford et al., 2000; Suzuki et al., 2006; Boldareva et al., 2008). This growth mode is likely shared by *Porphyrobacter* sp. HL-46 (Fuerst et al., 1993).

#### Metabolic analysis of unicyanobacterial consortia

**Figure 4:**
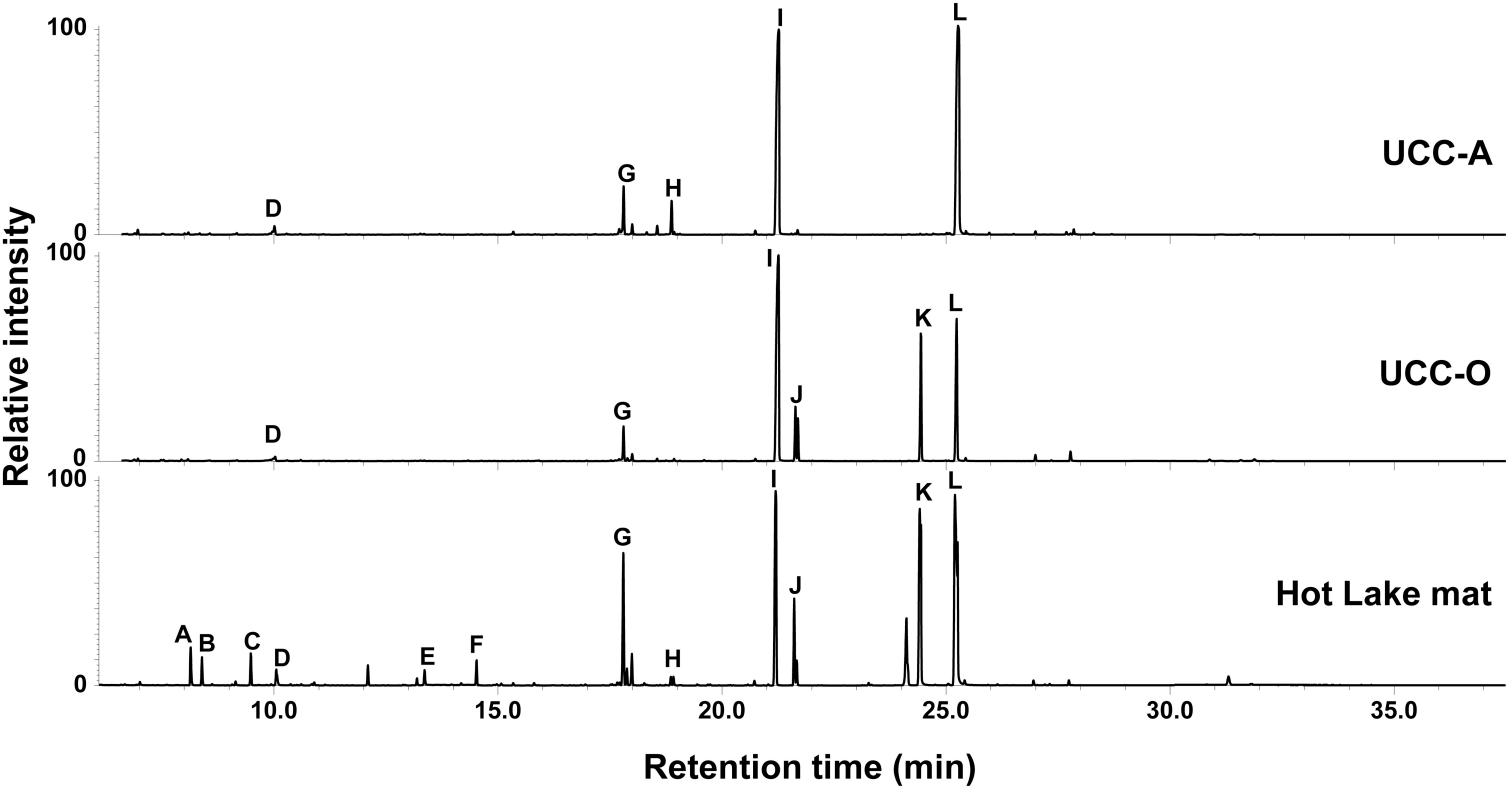
**Metabolic analysis of the consortia and the Hot Lake mat community**. GC-MS chromatograms depict polar metabolites identified by retention time and mass spectrum. Metabolite peaks are identified as carbonate ion (A), 3-hydroxybutyrate (B), 3-hydroxypentanoate (C), glycerol (D), pyroglutamate (E), glutamate (F), D-glucose (G), gluconate (H), glucosylglycerol (I), glucosylglycerate (J), sucrose (K), and trehalose (L).

As the consortia each possess a distinct mat-derived cyanobacterium coupled with the same suite of heterotrophic consorts, we performed a comparative metabolomic analysis of the intracellular metabolites present in the two consortia and the mat itself (Figure 4). Overall, the consortia exhibited many of the metabolites observed to be abundant components of the Hot Lake mat (sampled in mid-afternoon) at this resolution, though some clear differences were observed between the two consortia. The most common organic compounds in all three chromatograms were putative osmolytes: glycerol, pyroglutamate, glutamate, gluconate, glucosylglycerol, glucosylglycerate, sucrose, and trehalose (Schoor et al., 1995; Yancey, 2005). Glucose was also detected in all samples, as has been previously observed in cyanobacterial metabolomes (Krall et al., 2009; Narainsamy et al., 2011). Although glucosylglycerol and trehalose were abundant in both consortia, gluconate was a unique metabolite detected in UCC-A, and glucosylglycerate and sucrose were distinctive of UCC-O. Monomers (3-hydroxybutanoate and 3-hydroxypentanoate) of the bacterial carbon and energy storage polymers known as polyhydroxyalkanoates (PHA) were abundant in the mat sample but not in the consortia. The scarcity of PHA monomers in the consortia may reflect either the inability of most members to produce them, or that our culture conditions do not permit accumulation of extensive carbon and energy stores. Similar lower-abundance metabolites including amino acids (such as alanine, valine, isoleucine, proline, glycine, serine, threonine, phenylalanine, glutamic acid, and lysine), small organic acids (glyceric acid and lactate) and carbohydrates (e.g., ribose) were found in both consortia and the mat (data not shown). The similarity of metabolites found in these consortia to those in the mat further supported these consortia as reasonable model systems for the study of photoautotroph-heterotroph metabolic interactions relevant to the Hot Lake mat community.

### Structural dynamics of the assembling consortial biofilms

**Figure 5:**
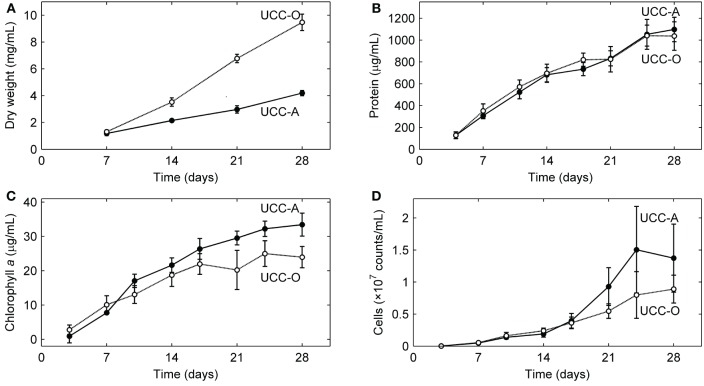
**Growth kinetics of the consortial biofilms**. Changes in biomass concentration of each unicyanobacterial consortium were measured as a function of time using **(A)** dry weight, **(B)** total protein, **(C)** chlorophyll *a*, and **(D)** flow cytometry (total cell counts). Values are averages calculated from triplicate biological replicates of three experiments (a total of nine samples). Error bars indicate 95% confidence intervals of the mean.

**Figure 6:**
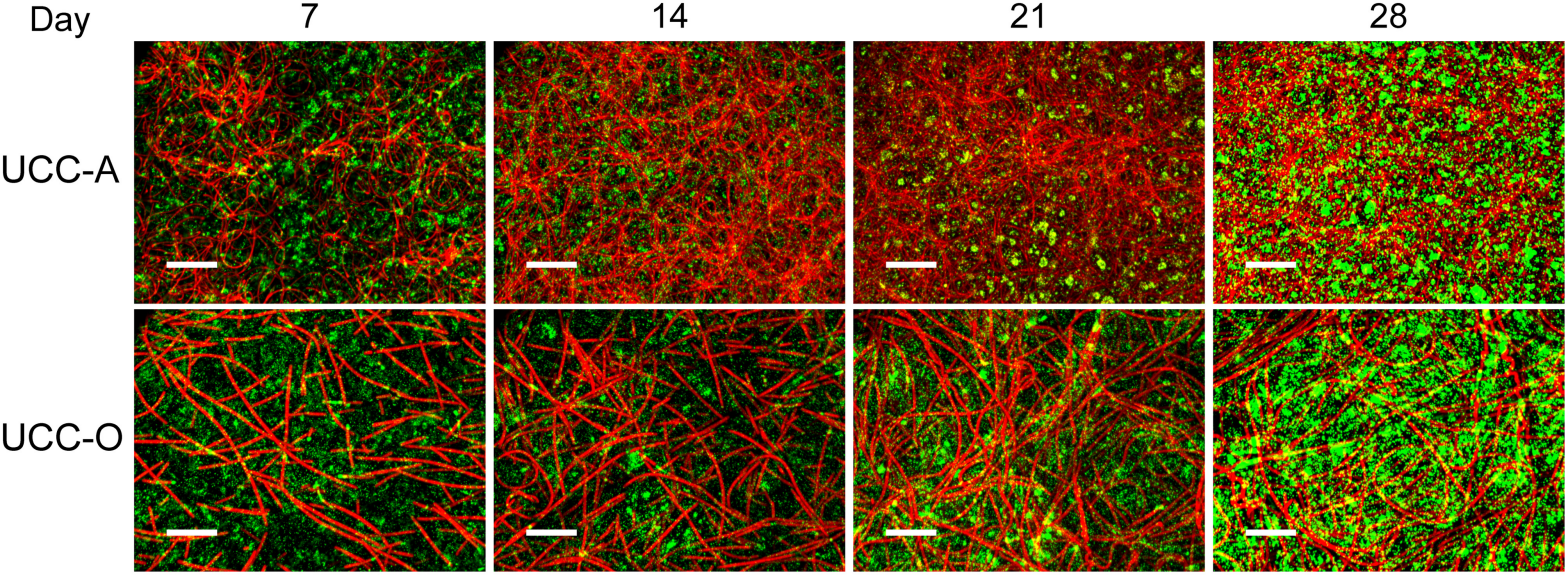
**Assembly of UCC-A and UCC-O biofilms**. Images are *z*-projections of stacks of images spanning the entire biofilm structure at a 1 μm step size and represent growth of the consortia on days 7, 14, 21, and 28. Chlorophyll *a autofluorescence* of the cyanobacteria is depicted in red and heterotrophs stained with SYBR Gold nucleic acid stain are depicted in green. The scale bars each denote 50 μm.

**Figure 7:**
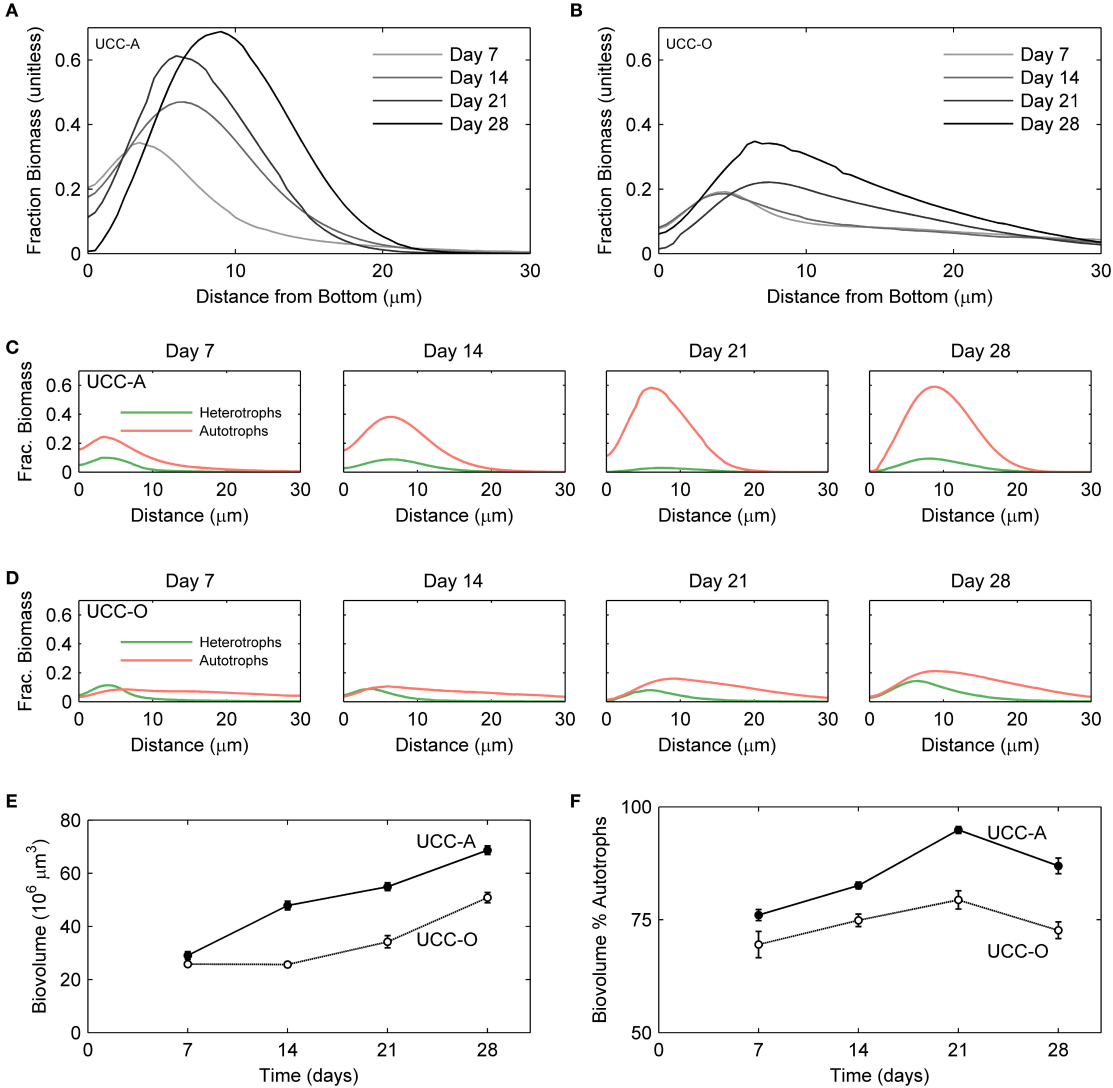
**Structural dynamics of autotroph-heterotroph balance in the consortial biofilms quantified by image analysis**. The fraction of the measured volume composed of cellular biomass as a function of depth is represented for UCC-A **(A)** and UCC-O **(B)**. Increasing darkness of the lines (from light gray to black), represent increasing biofilm age over the colonization period [7 (lightest gray), 14, 21, and 28 (black) days]. The fraction of volume composed of autotroph (red) and heterotroph (green) cellular biomass as a function of depth is represented for UCC-A **(C)** and UCC-O **(D)** at each time point. **(E)** Total biovolume of each consortium, plotted as a function of time. **(F)** Percent of each consortium's biovolume comprised by autotrophic biomass as a function of time.

We analyzed the growth kinetics of the assembling consortial biofilms over a period of 28 days. Because inorganic carbon was the sole carbon source supplied to the consortia, the cyanobacteria likely served as the sole primary producers of organic carbon for heterotrophic growth, as near neighbors of other organisms in the consortia are not known to fix carbon. Four metrics of biofilm development (dry weight, total protein, total chlorophyll *a*, and cell counts) all suggested linear (e.g., for total protein, UCC-A: *y* = 38.736*x* + 69.419, *R*^2^ = 0.9721; UCC-O: *y* = 35.978*x* + 126.09, *R*^2^ = 0.9368), rather than exponential, growth of the consortia (Figure 5). UCC-O produced approximately twice as much dry weight as UCC-A (Figure 5A) although the consortia contained similar amounts of protein (1097 ± 112 μg/mL and 1036 ± 130 μg/mL at day 28, respectively) (Figure 5B). As total protein has been extensively employed as a measure of total cellular biomass (e.g., Simon and Azam, 1989; Konopka et al., 1998), these data corroborate the microscopic observation (e.g., Figure 2) that UCC-O produced significantly more non-proteinaceous EPS matrix than UCC-A. The abundance of chlorophyll *a* provides an estimate of autotrophic biomass under non-light-limited conditions and increased equivalently with total protein until approximately day 17. Thereafter, chlorophyll *a* content of the consortia began to plateau, while total protein continued to increase linearly (Figures 5B,C), suggesting decreasing autotroph and/or increasing heterotroph growth rates after this point. Small heterotrophic cells that were free or loosely-associated with cyanobacterial filaments exhibited a linear increase for both consortia until day 14, after which cell counts for UCC-A showed exponential increase (Figure 5D). Cell counts are likely to be heavily biased toward detection of heterotrophs, as cyanobacterial filaments containing multiple cells are recorded by flow cytometry as one count. Microscopic image analysis (cf. Figures 6, 7), which quantifies heterotrophs tightly bound to EPS, also indicated that the two consortia experienced similar increases in heterotrophic biomass between days 14 and 28.

To understand the spatial organization and dynamics of autotroph-heterotroph balance within assembling biofilms, we coupled confocal microscopy to image analysis. Under our conditions, the nucleic-acid-dye SYBR Gold stained heterotrophs, but not cyanobacteria, allowing clear distinction between cyanobacterial and heterotrophic consorts. The growth of the biofilms is depicted in Figure 6 and quantitative analysis of their structural properties is presented in Figure 7. Consortial EPS was neither stained nor autofluorescent, so the volume occupied by EPS matrix could not be distinguished from void volume in our analysis. UCC-A formed a biofilm with higher biomass density (Figure 7A) than that of UCC-O and was composed of clusters of cyanobacterial filaments interspersed with microcolonies of heterotrophs (Figure 6, e.g., UCC-A day 21, cf. Figure 2A). The average final thickness of the UCC-A biofilms was 18.1 μm on day 28. In contrast, *Phormidium* sp. OSCR generated a thicker (23.0 μm on day 28), less cell-dense biofilm (Figure 7B); the motile filaments (and associated epibionts) of *Phormidium* sp. OSCR were more evenly dispersed throughout the biofilm (Figure 6) and were frequently observed gliding through the EPS matrix.

UCC-A and UCC-O displayed substantial differences in localization of heterotroph biovolume. Heterotrophs were colocalized vertically with the cyanobacteria in UCC-A at all time points, with their depths of maximal biomass averaging within 0.8 μm of each other (Figure 7C). Conversely, in UCC-O, cyanobacteria preferentially occupied the upper regions of the biofilm, with peak cyanobacterial biomass positioned 2.8 μm above peak heterotrophic biomass (Figure 7D). This statistically-significant difference (*p* < 0.005) between the consortia may be attributable to the phototactic behavior of *Phormidium* sp. OSCR that *P. priestleyi* str. ANA lacks (data not shown). Consistent with other metrics of growth (cf. Figure 5), both cyanobacterial and heterotrophic biomass generally increased with time in each consortium (Figures 7C,D). By day 28 the prevalence of heterotrophic biomass and the number and size of microcolonies had visibly increased in both consortia (Figures 6, 7C,D).

Quantitative partitioning of biovolume into autotrophic and heterotrophic fractions also revealed that the growth rate of cyanobacteria exceeded that of heterotrophs early in the biofilm's assembly. UCC-A displayed greater cellular biovolume than UCC-O at all time points, further suggesting the significantly larger dry weight of UCC-O is due to EPS production (cf. Figures 5A, 7E). UCC-O consistently supported greater heterotrophic biomass per unit cyanobacterial biomass than UCC-A (Figure 7F). In both consortia, however, cyanobacterial biomass represented >60% of the total biomass at all time points, peaking at 95% of UCC-A and 79% of UCC-O on day 21 before significantly declining on day 28. These data, combined with the plateauing chlorophyll *a* values (Figure 5C) and simultaneous increases in protein (Figure 5B) and cell counts (Figure 5D), suggest that by day 21 the growth rates of cyanobacteria in both consortia markedly declined, while those of heterotrophs substantially increased. Taken together, the quantification of biomass components and microscopic analysis support an early, autotroph-dominated phase of biofilm assembly, followed by a phase in which heterotrophic growth rates eclipse those of their primary producers.

#### Community dynamics of the assembling consortial biofilms

**Figure 8:**
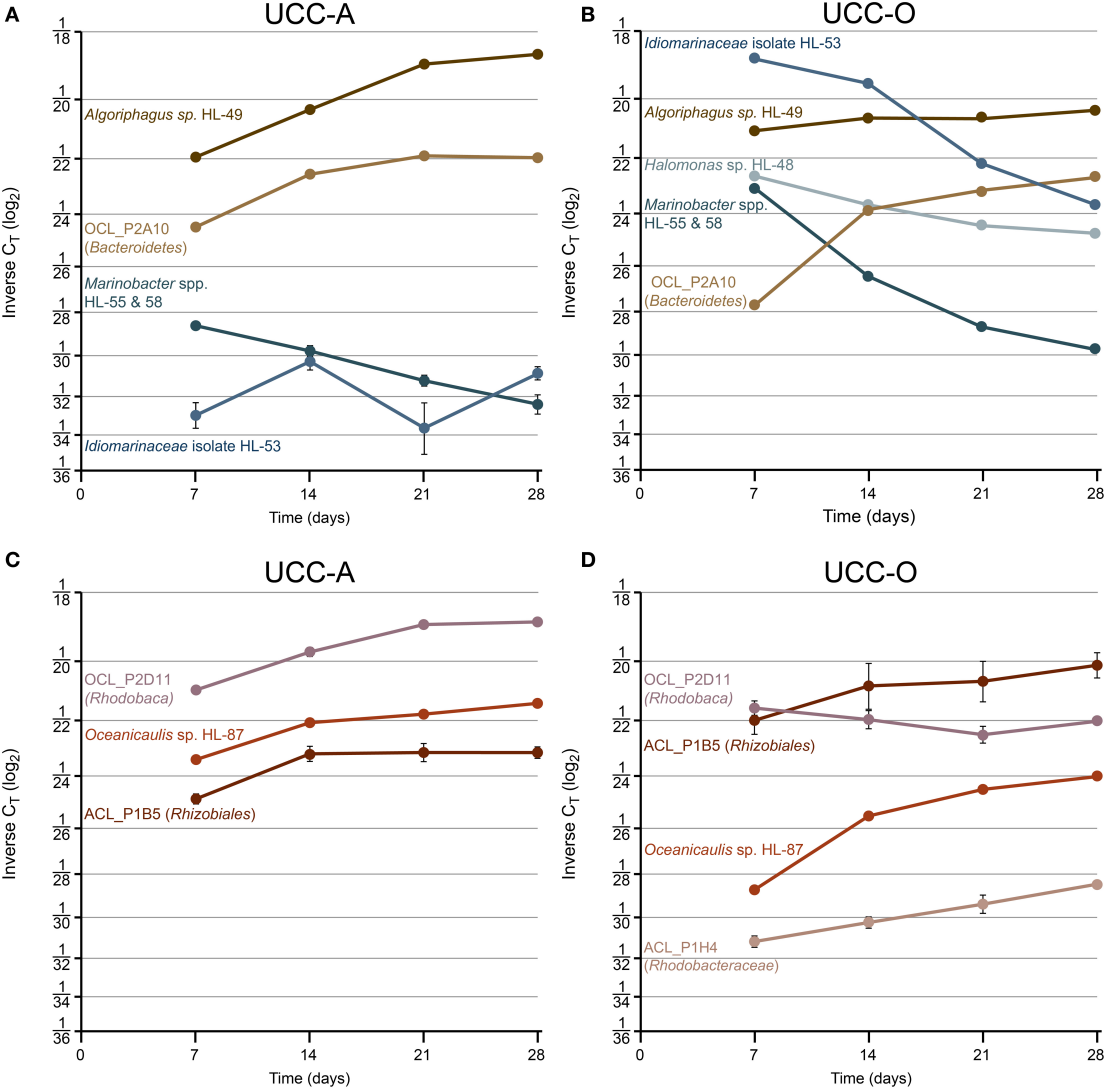
**Species-resolved dynamics in community structure within the assembling consortial biofilms**. Relative abundances of individual species quantified by real-time PCR at days 7, 14, 21, and 28. Variation in relative abundance of members of *Gammaproteobacteria* (shades of blue) and *Bacteroidetes* (shades of brown) (UCC-A, **A**; UCC-O, **B**) and *Alphaproteobacteria* (shades of red) (UCC-A, **C**; UCC-O, **D**) are depicted on a log_2_ scale. Average inverse *C*_*T*_-values and their 95% confidence intervals are presented for each signature, though in many cases the error bars are too small to be visible. Increasing inverse C_T_ signifies that the relative abundance of a given target sequence is increasing. *Halomonas* sp. HL-48 and *Rhodobacteraceae* sp. ACL_P1H4 were not detected in UCC-A.

To examine the contributions of the major consortium members to biofilm assembly, we used real-time PCR targeting of 16S rRNA genes to quantify the relative abundances of heterotrophic representatives of *Alphaproteobacteria, Bacteroidetes*, and *Gammaproteobacteria* over the growth period (Table 2). *C*_*T*_-values represent the PCR cycle at which an amplification plot crosses a given threshold and are, therefore, a measure of the concentration of target sequences in a reaction. Thus, lower *C*_*T*_-values indicate a larger number of target sequences. It is important to note that absolute abundances cannot be determined because the *rrnA* copy number of each member was unknown. However, comparisons of the abundances of each target between our consortia and across the time series are valid. We considered a *C*_*T*_ change >1 biologically significant, though statistical power generally permitted comparison over changes of less than 1 *C*_*T*_ (*p* << 0.05 by *t-*test assuming unpaired samples and unequal variance). The average *C*_*T*_-values and their standard deviations for each target within the consortia over the growth period are represented in Figure 8 and raw *C*_*T*_ data are supplied in Table S3.

Across all time points, gammaproteobacteria were much more common in UCC-O than in UCC-A, ranging from ~19- (*Marinobacter* spp.) to ~381-fold (*Idiomarinaceae* isolate HL-53) more abundant in UCC-O. In both consortia, populations of gammaproteobacterial species became increasingly less abundant or remained of low abundance over time (i.e., HL-53 in UCC-A). Initially-large relative abundances of *Idiomarinaceae* isolate HL-53 in UCC-O had diminished in abundance by nearly 50-fold by the end of the growth period, a trend that was also observed with the *Marinobacter* spp. HL-55 and HL-58 in both consortia. Because our probe recognized both *Marinobacter* spp. target sequences, these values should be interpreted as representing the contributions of both members (see Materials and Methods). *Halomonas* sp. HL-48 declined over five-fold in UCC-O during the growth period, whereas, in UCC-A, the population of *Halomonas* sp. HL-48 was below the limit of detection (Table S3). These data suggest a decreasing role for gammaproteobacteria in the consortia as assembly proceeded.

Members of *Bacteroidetes* grew rapidly in both consortia and became increasingly abundant with time as the communities matured. In contrast to the gammaproteobacteria, both members of *Bacteroidetes* were initially more abundant in UCC-A than UCC-O and exhibited stable or increasing relative abundances over the growth period. In UCC-A, both *Bacteroidetes* sp. OCL_P2A10 and *Algoriphagus marincola* str. HL-49 displayed approximately an order of magnitude increase in relative abundance. In UCC-O, similar increases were driven by a nearly 50-fold gain in the relative abundance of *Bacteroidetes* sp. OCL_P2A10, the majority of which was observed early in the growth period. *Algoriphagus marincola* str. HL-49 did not significantly change in relative abundance in UCC-O.

Whereas members from *Bacteroidetes* and *Gammaproteobacteria* generally demonstrated similar trends in relative abundance between consortia, members of *Alphaproteobacteria* exhibited distinct population dynamics between UCC-A and UCC-O. *Rhizobiales* sp. ACL_P1B5 exhibited nearly equivalent, moderate (approximately four-fold) increases in relative abundance in both consortia. Conversely, though *Oceanicaulis* sp. HL-87 was initially about five-fold more abundant in UCC-A, this organism gained in relative abundance more rapidly in UCC-O (~40-fold) than in UCC-A (~4-fold). *Rhodobaca* sp. OCL_P2D11 increased significantly more rapidly in relative abundance in UCC-A, remaining stable or slightly diminishing in UCC-O over the growth period. *Rhodobacteraceae* sp. ACL_P1H4 could not be detected in UCC-A and was of low but increasing abundance in UCC-O. This may indicate that, under our biofilm assembly conditions, this strain may fill a niche in UCC-O that either does not exist or is occupied by another member in UCC-A. In general, the divergence in the abundance patterns of alphaproteobacterial members may reflect distinct interactions with each of their cyanobacterial partners.

Despite the fact that the consortia share essentially identical heterotrophic memberships, these data suggest that the relative abundances of individual heterotrophs in the assembling consortial biofilms could be significantly impacted by the specific cyanobacterium serving as its primary producer. However, similar overall trends in assembly could be observed in both consortia over the growth period (i.e., decrease in the four members of *Gammaproteobacteria* and increase in the two members of *Bacteroidetes* with time), which may suggest that co-varying members may perform similar, complementary, or linked metabolic functions in the community. Altogether, these data support the likelihood that the consortia contain a spectrum of autotroph-heterotroph (and, subsequently, heterotroph-heterotroph) interspecies metabolic interactions that may range from the mutualistic to the opportunistic.

## Discussion

Laboratory model systems can be utilized to investigate ecological principles applicable to microbial ecosystems (reviewed in Jessup et al., 2004). Although phototrophic biofilms have recently been studied in-depth under flow conditions (Van der Grinten et al., 2004; Congestri et al., 2006; Buhmann et al., 2012; Pippo et al., 2012; Larson and Passy, 2013), these studies have generally focused upon the photoautotrophic members and paid little attention to the contributions of heterotrophic consorts to biofilm formation. Though some recent attempts to identify and localize heterotrophs in the primary succession of phototrophic biofilms have been made (Roeselers et al., 2007; Buhmann et al., 2012), the metabolic roles of individual heterotrophs, their interactions with the primary producer(s) and each other, and their contributions to community productivity and stability have not yet been elucidated.

Because the consortia are constrained by cultivation in closed vessels, changes in their community structures reflect dynamicity in nutrient availability, substrate utilization, and interspecies interactions as assembly progresses and total biomass increases. Communication of the consortia with the broader environment was limited to gas exchange and light energy input, allowing these consortia to serve as microecosystem models in which organisms and metabolites are physically retained within the system (Gordon et al., 1969). Therefore, the relative abundance of each species over time is solely determined by its growth rate (less its turnover rate), compared with the net growth rates of the other members under the prevailing conditions of the assembling community.

Growth of autotrophs in the consortia drives community biomass production by providing the organic carbon that, in turn, supports increasing growth rates of heterotrophic consorts. Primary producer growth is expected to lead heterotroph growth in the early succession of assembling autotrophic communities (Odum, 1969), as little-to-no organic carbon is likely to be initially available (Fierer et al., 2010). Organic carbon may become available to heterotrophs in diverse forms, such as exuded low-molecular-weight photosynthate, EPS, or cell detritus. Furthermore, as only trace ammonium is available within the culture medium, the energy-intensive reductions of nitrate or dinitrogen are the only processes by which the community can assimilate nitrogen. It is likely that many of our heterotrophic consorts lack the ability to assimilate nitrate into biomass, but most cyanobacteria perform this function (Ohashi et al., 2010; Luque-Almagro et al., 2011). Therefore, it is probable that the cyanobacteria in our consortia also serve as the major entry point for reduced nitrogen species into the community early in the growth period, as they are unlikely to be energy-limited under the continuous light regime. Cyanobacterial growth is likely to be nutritionally limited in the consortia by bioavailable phosphate, especially considering the sparing solubility of magnesium phosphates. Although cyanobacterial growth leads biofilm assembly in our system, we cannot rule out an initial role for heterotrophs in facilitating early attachment of cyanobacteria to the surface, as has been previously reported under advective flow (Roeselers et al., 2007).

The assemblages of heterotrophic phylotypes within the consortia do not appear to have been retained by stochastic processes alone. Prior to experimentation, each consortium experienced significant sequential dilution (>>1 × 10^20^-fold from the inoculum), such that the heterotrophs present can be considered to have successfully competed for and occupied niches within these consortia. As the consortia were continuously exposed to moderate photon flux (~20 μE/m^2^/s), it is probable that the cultures remained perpetually oxic; consequently, it is unlikely that anaerobic microniches existed during enrichment. Within the niche space provided by the consortia (e.g., permitting only aerotolerant members), it is likely that the initial relative abundances of each species impacted the ability of the organisms to compete for and occupy niches (i.e., priority effects). Therefore, it cannot be assumed that the heterotrophs retained in the consortia are necessarily the strongest competitors, of all heterotrophic organisms within the mat, in the niches they occupy. However, as the heterotrophs within the Hot Lake mat likely rely upon cyanobacterial primary production for organic carbon, it is not surprising that the most abundant mat members would also be adapted for life in close association with cyanobacteria in culture. The majority of the heterotrophic consorts are representatives of OTUs abundant within the Hot Lake mat community (Lindemann et al., 2013) and all are assigned to taxa frequently found in hypersaline cyanobacterial mats (e.g., *Halomonas, Marinobacter*, and *Rhodobacteraceae*; Jonkers and Abed, 2003). Phylogenetic near neighbors of these members have been frequently observed as epibionts of *Trichodesmium* consortia in the open ocean (Hmelo et al., 2012), cyanobacterial aggregates from the Baltic Sea (Tuomainen et al., 2006), and isolates from cyanobacterial cultures (Choi et al., 2009; Hube et al., 2009; Shi et al., 2009). Furthermore, our consortia contain multiple members of the *Roseobacter* clade of *Alphaproteobacteria*, which are known to be frequent members of phototrophic mats and consorts of marine photoautotrophs (Buchan et al., 2005; Brinkhoff et al., 2008; Mayali et al., 2008; Wagner-Döbler et al., 2009). We therefore propose that the Hot Lake unicyanobacterial consortia are generalizable model systems for the study of cyanobacteria-heterotroph interactions relevant to the Hot Lake microbial mat and to phototrophic mats broadly.

The retention of the same heterotrophs in both consortia suggests that the niches generated by each cyanobacterium's autotrophic growth and secondary heterotrophic metabolisms are similar enough to permit the employment of analogous metabolic strategies. This similarity is further emphasized in that heterotrophic taxa exhibit similar relative abundance trends in both consortia over the growth period, such that members from *Bacteroidetes* and *Alphaproteobacteria* increase in abundance with a concomitant reduction in members of *Gammaproteobacteria.* These larger-scale patterns lead us to hypothesize that the members of these taxa within the consortia may share some overarching metabolic or physical traits (e.g., in specificity of substrate utilization, growth efficiency per unit substrate, chemotaxis toward or physical attachment to cyanobacteria) that are either advantageous or disadvantageous at certain points in biofilm assembly. If true, the similar trends in relative abundance at higher taxa may indicate that niches within the two consortia develop in broadly similar ways as these communities assemble, independent of which cyanobacterium serves as its primary producer.

These similar assembly patterns contrast with the large differences in relative abundances of specific members (e.g., hundreds-fold within *Gammaproteobacteria*) between the consortia, suggesting divergence in the metabolic opportunities afforded by each cyanobacterium. Disparity in the identities and relative abundances of intracellular metabolites between consortia implies that distinct metabolic processes are operative in each, driving differences in carbon partitioning between them. This is especially evident in the large osmolyte pools required for growth under hypersaline conditions that require large investments of resources by both cyanobacteria and heterotrophs. Another large difference in carbon resource availability might be due to the extracellular polysaccharides produced by each cyanobacterium; both sugar composition and linkage structure could differ between the cyanobacteria, thereby requiring different glycosyl hydrolases for heterotrophic consumption. These differences in the availability and speciation of carbon or nitrogen resources likely drive the distinct growth patterns observed at the species level between the two consortia. Variation in the relative abundance and growth kinetics of specific heterotrophs between the two consortia strongly suggests that the web of autotroph-heterotroph and heterotroph-heterotroph interactions operating within each assembling consortium generates niches that, while similar, differ enough to form and maintain disparate community structures.

The metabolic capacities of phylogenetic near neighbors permit tentative assignment of hypothetical functional roles of heterotrophs within the consortia. Experimental and genomic evidence suggest that near neighbors of the consortia's gammaproteobacteria (i.e., members of *Idiomarinaceae* and *Marinobacter*) are specialists in peptide and amino acid degradation, but lack the ability to use most carbohydrates as carbon sources (Donachie et al., 2003; Hou et al., 2004; Ivanova et al., 2004; Bowman and McMeekin, 2005). The relative loss of these gammaproteobacteria over the colonization period may be due to the sparing availability of extracellular peptides. *Halomonas* sp. HL-48 is likely more nutritionally versatile than the other gammaproteobacteria (Garrity et al., 2005), which may explain why its relative decline in abundance within UCC-O is less severe. As our data suggest that a significant amount of carbon is stored in non-proteinaceous EPS, especially in UCC-O, organisms capable of hydrolysis and utilization of these polymers as carbon sources are likely to perform well in the consortia. Members of *Bacteroidetes*, including the genus *Algoriphagus* (Alegado et al., 2011), are known to specialize in degradation of biopolymers, such as polysaccharides (Gómez-Pereira et al., 2012). Their genomes tend to be rich in genes encoding peptidases, glycoside hydrolases, receptors and transporters for high-molecular-weight compounds, as well as adhesion proteins that facilitate attachment to and degradation of biomass (Fernández-Gómez et al., 2013). A lifestyle focused upon the consumption of EPS is consistent with the increasing abundances we observed in members of *Bacteroidetes* in both consortia. As a whole, the nutritional diversity and apparent novelty of members of *Alphaproteobacteria* make it difficult to propose an overall metabolic strategy for this group, and it is possible that each organism pursues an idiosyncratic strategy. However, some of our alphaproteobacteria are representatives of genera known to contain AAPs (*Rhodobaca*, *Roseibacterium*, and *Porphyrobacter*), and therefore may represent a second means by which light energy can enter the consortia.

Considering their ubiquity and diversity, the primary succession of microbial systems has received relatively little attention to date (Fierer et al., 2010). This is, in part, due to the complexity of natural microbial communities and our inability to control the environmental parameters that govern them. The reduced complexity of the unicyanobacterial consortia described herein and their members' taxonomic similarity to frequently-observed consorts of cyanobacteria and microalgae make them useful model systems in which to study the dynamic photoautotroph-heterotroph interactions driving phototrophic community assembly. Additionally, the capacity for comparative analysis afforded by consortia with distinct autotrophs, but shared heterotrophic membership, lends further potential for the niche-resolved study of microbial primary succession under variable environmental conditions. Although the consortia exhibit very distinct community structures, we hypothesize that they may maintain similar relative abundances of functional genes as their biofilms assemble because niches recruit organisms based upon their functional capacity (Kassen and Rainey, 2004). Determination of each consortium's metabolic capacity through metagenomic sequencing and attribution of these capacities to individual member genomes (Wrighton et al., 2012) will greatly aid in dissecting out the potential functional roles played by each member within the consortia. Furthermore, such metagenome-enabled metabolic reconstruction of each species will shed light on the principles governing recruitment, maintenance, compartmentalization, and redundancy of metabolic function (Gudelj et al., 2010; Johnson et al., 2012) in assembling phototrophic communities.

### Conflict of interest statement

The authors declare that the research was conducted in the absence of any commercial or financial relationships that could be construed as a potential conflict of interest.

## Supplementary material

Figure S1 **Light micrographs of the two Hot Lake unicyanobacterial consortia, UCC-A (A) and (B) UCC-O**. The scale bar denotes 10 μm.
