# Supplementary figures and images for: Phototrophic biofilm assembly in microbial-mat-derived unicyanobacterial consortia: model systems for the study of autotroph-heterotroph interactions
Source: Front Microbiol. 2014 Apr 7;5:109. doi: 10.3389/fmicb.2014.00109 (PMC3985010; doi:10.3389/fmicb.2014.00109)

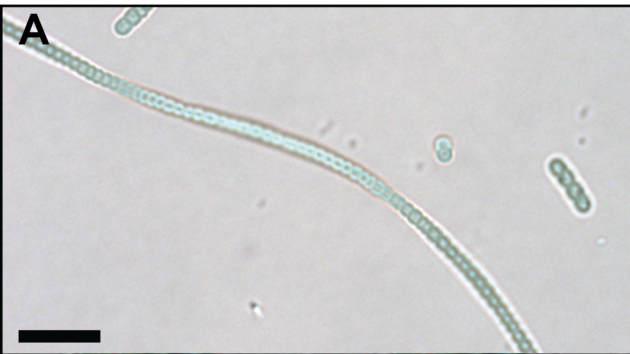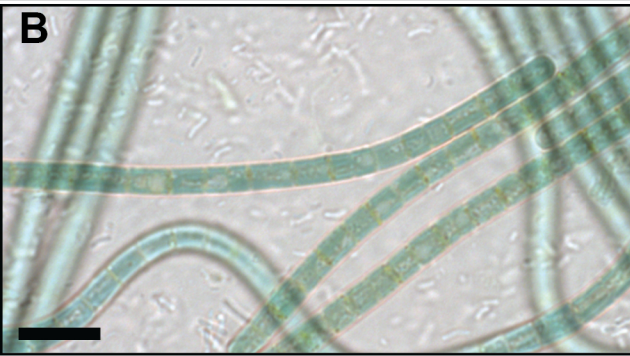

Supplement: Figure S1 — Light micrographs of the two Hot Lake unicyanobacterial consortia, UCC-A (A) and (B) UCC-O. The scale bar denotes 10 μm. [file Presentation1.PDF]
