# Supplementary material for: Phototrophic biofilm assembly in microbial-mat-derived unicyanobacterial consortia: model systems for the study of autotroph-heterotroph interactions
Source: Front Microbiol. 2014 Apr 7;5:109. doi: 10.3389/fmicb.2014.00109 (PMC3985010; doi:10.3389/fmicb.2014.00109)
Supplement: Supplementary file 2 [file DataSheet1.PDF]

Supplemental Table 1. GenBank accession numbers associated with this study

Sequence	Accession
ACL_P1H4	KJ004400.1
HL7711_P4E7	KJ004401.1
OCL_P2D11	KJ004402.1
ACL_P1B5	KJ004403.1
OCL_P2A10	KJ004404.1
HL7711_P5D1	KJ004405.1
ACL_P2D9	KJ004406.1
OCL_P2H12	KJ004407.1
OCL_P1F4	KJ004408.1
OCL_P1H5	KJ004409.1
ACL_P1H5	KJ004410.1
OCL_P2G9	KJ004411.1
OCL_P1H8	KJ004412.1
HL-46	KJ004413.1
OCL_P1H2	KJ004414.1
